# Membrane Fouling Control in Water Treatment

**DOI:** 10.3390/membranes12060551

**Published:** 2022-05-25

**Authors:** Yi-Li Lin

**Affiliations:** Department of Safety, Health and Environmental Engineering, National Kaohsiung University of Science and Technology, Kaohsiung 824, Taiwan; yililin@nkust.edu.tw

The stress of freshwater scarcity has become a severe problem worldwide and drives the development of technologies for water recycling and reuse. Among these technologies, membrane separation has received a great amount of attention because of its simple operating procedure, few chemical additions, and broad removal of pollutants of different sizes. However, for both conventional pressure-driven membrane separation (such as nanofiltration (NF) and reverse osmosis (RO)), and the emerging concentration-driven forward osmosis (FO) processes, the major challenge for the practical application of various membranes is the inevitable fouling, which can cause drawbacks by increasing cleaning frequency and operating cost but reducing membrane life. Therefore, fouling control in the development of membrane technology is crucial, especially for the treatment and recycling of various water sources with complicated matrices.

The aim of this Special Issue “Membrane Fouling Control in Water Treatment” was to shed light on the latest advances of membrane fouling control techniques, such as in-situ membrane surface modification, incorporation of emerging nanocomposites or functional copolymer into membrane material, and fouling mechanism illustration. Here are the key findings of the contributors in this Special Issue.

Wang et al. [1] prepared polyethylene glycol (PEG) non-covalent-functionalized multi-walled carbon nanotube (MWCNT) membranes through vacuum filtration, which greatly enhanced membrane hydrophilicity, with better removal of humic acid (HA) and lower transmembrane pressure (TMP) growth compared to a commercial 0.01-μm PVDF ultrafiltration (UF) membrane. Moreover, the PEG-MWCNT membrane exhibited excellent antifouling ability with 79.4% TMP recovery through flushing, which effectively prolonged the service life of membrane.

Wang and Ding [2] used self-made TiO_2_ nanoparticles to modify a UF membrane (PVDF-2) to increase membrane hydrophilicity and reduce surface pollution in the A/O-MBR (anoxic–aerobic membrane bioreactor) process. The modified membrane exhibited significantly improved antifouling performance and was successfully applied to the treatment of landfill leachate.

Lin et al. [3] modified in-situ a commercial nanofiltration membrane, NF90, through the concentration-polymerization-enhanced radical graft polarization method by applying two agents of 3-sulfopropyl methacrylate potassium salt (SPM) and 2-hydroxyethyl methacrylate (HEMA). The modified membranes exhibited considerably enhanced separation performance of NaCl rejection and pharmaceutical and personal care product (PPCPs) rejection than the pristine membrane. Moreover, the modified membranes exhibited relatively less flux decline and higher percentage of reversible fouling than the pristine membrane when treating the feedwater with a high silica concentration, and the fouling mechanism was confirmed to be intermediate blocking of membrane pores. Overall, the in-situ modification technique with a low monomer concentration is cost-effective and easily performed in practical applications for mitigating silica fouling, as well as improving NaCl and PPCP rejection. 

Li et al. [4] blended amphiphilic zwitterion polysulfone-co-sulfobetaine polysulfone (PSf-co-SBPSf) copolymer to prepare antifouling PSf UF membranes. The PSf/PSf-co-SBPSf blend membranes show significant increases in porosity, water permeance and surface hydrophilicity and have excellent antifouling abilities and thermostability. Therefore, the PSf/PSf-co-SBPSf blended membranes have promising potential for high-temperature separation application.

Ma et al. [5] systematically evaluated the fouling mechanism of an anion exchange membrane (AEM) induced by different natural organic matter (NOM) and calcium via the extended Derjaguin–Landau–Verwey–Overbeek (xDLVO) approach. The results show that the presence of calcium ions can form Ca–NOM complex on AEM, and short-range acid–base (AB) interaction energy played a significant role in the compositions of interaction energy during the electrodialysis (ED) process, which was a dominating indicator for evaluating the tendency of AEM fouling by NOM.

The research results and critical discussion from these five contributions can provide state-of-the-art knowledge that fits the gap for future developments in the field of membrane fouling control. My sincere thanks to all the contributors for the successful publication of this Special Issue.
